# How Do Trematodes Induce Cancer? A Possible Evolutionary Adaptation of an Oncogenic Agent Transmitted by Flukes

**DOI:** 10.1111/eva.70070

**Published:** 2025-01-22

**Authors:** Péter Apari, Gábor Földvári

**Affiliations:** ^1^ Independent Researcher Szekszárd Hungary; ^2^ Institute of Evolution HUN‐REN Centre for Ecological Research Budapest Hungary; ^3^ Centre for Eco‐Epidemiology National Laboratory for Health Security Budapest Hungary

**Keywords:** cancer, *Clonorchis sinensis*, evolution, oncogene, *Opisthorchis viverrini*, parasite, *Schistosoma haematobium*, trematodes, tumour

## Abstract

There is strong epidemiological evidence that development of various cancer types is linked to infection with flukes (Platyhelminthes: Trematoda) in Africa, Asia and the Middle East. The exact nature of the mechanism by which cancer is induced by these parasites is unknown. Here, we provide a new hypothesis suggesting that flukes are not the primary cause of cancer but act as vectors of cancer‐inducing microbial pathogens. These pathogens adaptively induce tumours to attract and help flukes to feed on blood from the tumour. Pathogen take‐up by fluke vectors also takes place in the tumour; therefore, tumour formation in this case is the result of a mutualistic and adaptive relationship between the microbe and the helminth parasite. The suggested mechanism for cancer induction provided here may help us gain deeper understanding about cancer in general and its relationship with microbes and parasites. By further elaborating the unique nexus between flukes, carcinogenic microbes and cancer, in the future it will also help us to broaden our oncological perspective to reduce human death and suffering from this serious disease group.

## 1 Introduction

Current global changes increase contacts between neglected tropical parasites and people, leading to serious public health problems. Both experimental and epidemiological evidences strongly implicate infection with three different fluke species (*Schistosoma haematobium*, *Opisthorchis viverrini* and *Clonorchis sinensis* ) in cancer development, and these parasites are considered Group 1 (extensively proven) carcinogenic agents. However, limited information exists on how these parasites induce host tumours. According to the current paradigm, the main reason is chronic inflammation, considered a hallmark of cancer (Brindley and Loukas 2017). Here, we challenge this view by suggesting that the cause of carcinogenesis is probably an oncogenic pathogen (virus or bacterium) transmitted by these parasitic worms. Published evidence indicates that flukes can be vectors of pathogenic bacteria (*Neorickettsia* sp.), and these bacteria circulate among trematodes both vertically and horizontally, (Gibson et al. 2005; Deenonpoe et al. 2017). Moreover, according to intriguing new research, the fluke species *O. viverrini* was found to be linked to the known oncogenic bacterium *Helicobacter pylori* , which is a similar host–vector–bacterium system. Because of convergent evolution (Sackton and Clark 2019), this can be a common feature that opens new perspectives and a possible paradigm change in both parasite biology and human oncology (Deenonpoe et al. 2017). We hypothesize that carcinogenesis is adaptive for the microbial agent because it enhances its horizontal transmission to other flukes within the same host. Adult flukes feed on the tumour itself, which is rich in blood vessels, and thus flukes provide an attractive target for an oncogenic agent to spread. The geographical distribution of some diseases provides indirect support for our hypothesis. Taking these flukes as an example, they are known to cause bile duct cancer, probably linked to *H. pylori* , in tropical areas where they are endemic (Deenonpoe et al. 2017). On the other hand, in temperate regions where the fluke vectors are absent, *H. pylori* is linked with gastric cancer and mucosa‐associated lymphoid tissue lymphoma (MALT) (Ohnishi et al. 2008). This indicates different evolutionary histories and different ecological relationships of the two distinct bacterial strains.

## 2 Does Inflammation Really Matter?

Interestingly, besides some pathogenic microbes, chemical substances and radiations, three fluke species (*S. haematobium*, *O. viverrini* and *C. sinensis* ) are considered Group 1 carcinogenic agents (Brindley and Loukas 2017). Infection with *S. haematobium* has the highest risk of developing bladder cancer, mainly in the Middle East and some parts of the African continent, while *O. viverrini* and *C. sinensis* are linked to bile duct cancer, especially in Southeast Asia and China (Correia da Costa et al. 2014). The pathogenesis, however, of chronic infection with these flukes leading to the above‐mentioned tumours is unclear to the present day. According to the most widely accepted hypothesis, the continuous irritation and tissue damage caused by the adult flukes and their eggs lead to chronic inflammation, which is considered a hallmark of cancer (Brindley and Loukas 2017). Moreover, scientists found evidence that there are possible oncogenes directly linked to flukes, and this seemingly strongly supports the fluke‐induced cancer hypothesis (Chaiyadet et al. 2022). However, studies did not investigate whether these supposed virulence genes somehow interact with the fluke microbiome. Without this information, we cannot rule out that the flukes contributed to the development of cancers as vectors of oncogenic pathogens (Dheilly et al. 2019).

There are two medically more important species of *Schistosoma* blood flukes beside *S. haematobium*, namely *Schistosoma mansoni* and *Schistosoma japonicum*. All three are responsible for chronic inflammation, but only *S. haematobium* is a Group 1 carcinogenic agent. The other two are not linked to the development of cancer; however, they live in different type of tissues inside the human host that might have different susceptibility for the disease (von Bülow et al. 2021). There is more convincing experimental evidence against the inflammation hypothesis. Scientists inserted the oncogenic virulence gene cagA of *H. pylori* into mice. Although all of the cells of the transgenic experimental mice were expressed, only gastric cancer and MALT developed without inflammation (Ohnishi et al. 2008). *Helicobacter pylori* can be cagA positive or negative and can cause gastric cancer and MALT depending on the status of the oncogenic virulence genes (Tserentogtokh et al. 2019). Moreover, there are potential molecular mechanisms involving CagA protein which is responsible for the development of cancer (Hatakeyama 2017). Scientists were surprised by the results and the fact that inflammation may be not so essential in the development of cancer as previously thought (Ohnishi et al. 2008). How can we explain that in all other experiments the researchers experience the appearance of inflammation but not in this case? The speciality of this experiment is that bacteria are not present, but only bacterial oncogenes. Inflammation is a typical immune reaction against various pathogens, even cancer‐causing ones, but probably not against bacterial oncogenes. It is important to mention that virus oncogenes regularly induce inflammation probably because insertion of viral oncogenes into the host genome is part of the natural viral life cycle which is not true for bacterial oncogenes (Varn et al. 2018). Moreover, inflammation is also paradoxical phenomenon in relation to cancer because in some cases it supports while in others it inhibits the development of tumours (Liu et al. 2023). The former can happen because occasionally the inflammatory responses are diverted for the interests of the tumours not for the host organism. Based on this, we suspect that development of cancer is not a by‐product but probably a direct adaptation of the bacterial pathogen. For this reason, there has to be an evolutionary advantage for bacterial populations that induce cancer.

## 3 Flukes, Bacteria and Tumours Are Interconnected

The fascinating studies linking a Southeast Asian liver fluke *O. viverrini* to *H. pylori* open the door for new speculations about the connection between these parasites and bile duct cancer (Deenonpoe et al. 2017). We hypothesize that flukes are not the primary cause of cancer but vectors of oncogenic viruses or bacteria, and the tumour is adaptive for the pathogens to spread horizontally and infect new fluke vectors within the same host. In particular, hepatitis B virus and hepatitis C virus are now accepted causes of cholangiocarcinoma and are known to interfere with barriers to cancer (Ewald and Swain Ewald 2014).

Although the bacteriome of several trematodes has now been fully characterized, via sequencing of the 16S rRNA bacterial gene, including some flukes with a similar life cycle to *Opisthorchis*, there are some methodological shortcomings (Jorge, Dheilly, and Poulin 2020; Jorge et al. 2022; Salloum, Jorge, and Poulin 2023). Pakharuhova et al. (2023), for example, failed to find *Helicobacter* spp. in *O. viverrini* , *C. sinensis* and *Opisthorchis felineus* (Pakharukova et al. 2023). Their method, however, had major limitations, as it was based on amplification of the 16S ribosomal RNA gene using nonspecific bacterial primers. Unfortunately, the minimal number of PCR cycles was not enough to identify all low‐abundance bacterial species; it therefore does not exclude the presence of *H. pylori* in low copy numbers. In fact, a key study (Thanaphongdecha et al. 2022) presented novel evidence that L‐fucose, which is detected in the gut epithelium and tegument of *O. viverrini* , plays a role in the adhesion of *H. pylori* to this liver fluke. They demonstrated that the binding of *Helicobacter* to the gut of *O. viverrini* is fucose‐dependent. As a consequence, the liver fluke *O. viverrini* can be considered a reservoir of *H. pylori* , and fucose is a selective molecule for cagA‐positive *H. pylori* binding in the worm's gut, which can promote *H. pylori* colonization and enhance the biliary pathogenesis and carcinogenesis. This experimental evidence has three important implications. First, the relationship between the parasitic worm and the bacterium may be mutualistic. Second, the worm has an advantage from harbouring only cancer‐inducing (cagA‐positive) *H. pylori* that implies carcinogenesis has an advantage for both mutualistic partners. Third, the specific binding of the bacterium in the fluke gut is linked with the fluke's feeding on tumours.

**FIGURE 1:**
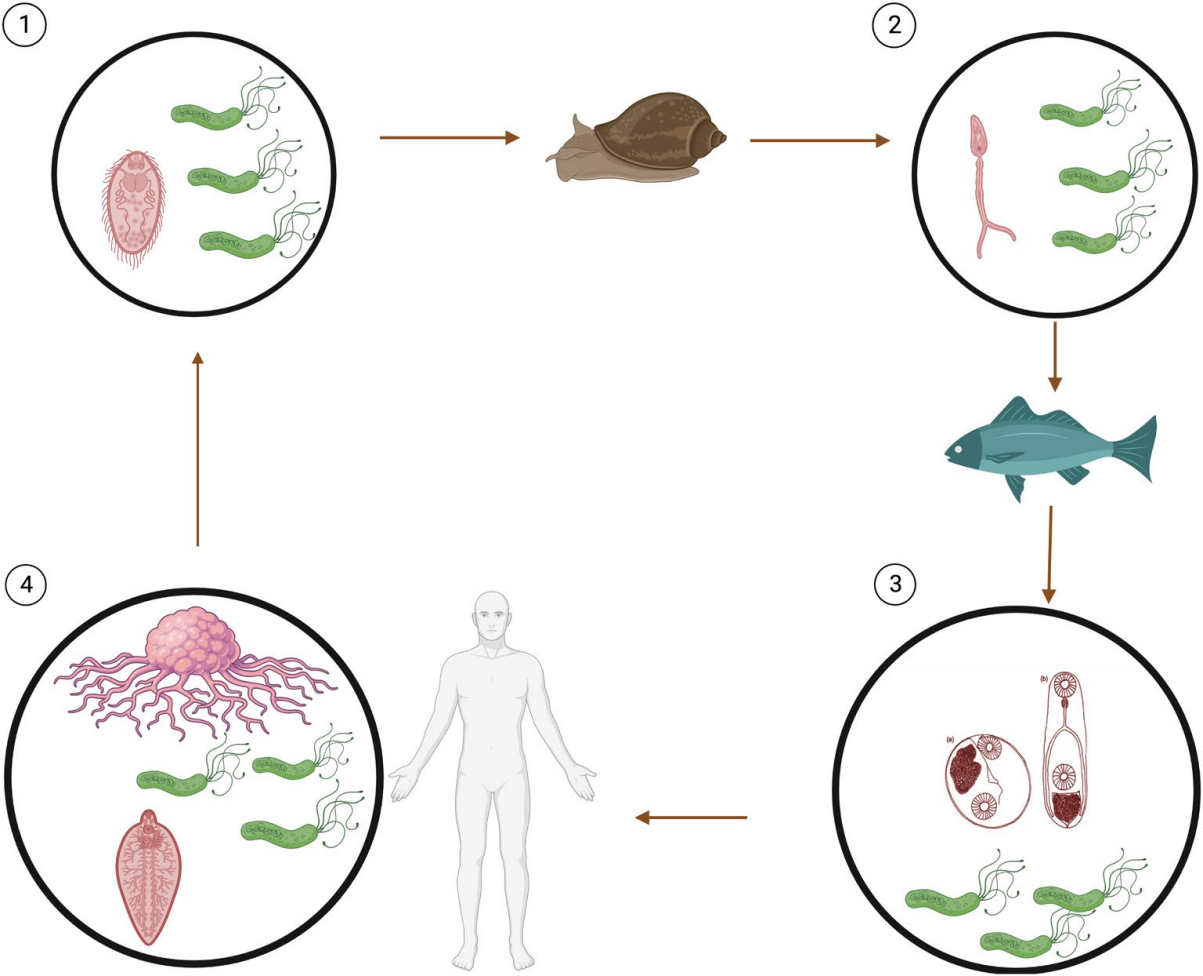
The life cycle of the parasitic fluke *Opisthorchis viverrini* and its suggested role in oncogenesis. Fully developed eggs are passed in the faeces and ingested by snails. In the snails, miracidia (1) are released and undergo various stages of development, including sporocysts and rediae (not shown) and, finally, cercariae (2). These cercariae are then released from the snail and can penetrate the skin of various fish where they encyst and become metacercariae (3, (a) encysted or (b) excysted) in the tissues of the fish (Waikagul and Thaenkham 2014). Humans become infected by ingesting undercooked fish containing the infective metacercariae. We hypothesize that the symbiotic *Helicobacter pylori* bacteria (shown as green flagellated cells) are being transmitted without difficulty through all life stages of the parasitic fluke. Infection with these flukes adaptively cause tumours to attract multiple flukes and infect them successfully with the bacteria (4). Created with BioRender.com. ‘Reprinted from (Waikagul and Thaenkham 2014) with permission from Elsevier’.

It is well known that vascularization is essential for the tumours to grow and survive (Folkman 1971). Because parasitic flukes are blood feeders (Feldmeier et al. 2016), they have the capability to detect and move towards tissues richest in blood vessels and follow the increased temperature of the targeted host organism (Bryant and Hallem 2018). Published evidence shows that some pathogens can make the host attractive also for vectors to adaptively increase effective spreading in the population (Zhang et al. 2022). Tumours have abundant blood vessels and contain large amounts of blood, which is attractive for flukes because tumours have a higher basal temperature compared to normal tissues (Knapp et al. 2022). The tumour is probably full of particles and cells of the oncogenic agents, which can infect multiple new flukes and spread horizontally in this way. However, it is well known that parasitic trematodes have a very complex life cycle and many different developmental states (Benesh, Parker, and Chubb 2021). For the vector role, it is essential that the oncogenic agents spread vertically too, through these diverse developmental states. There are convincing examples where trematode species are indeed vectors of pathogenic bacteria called *Neorickettsia* spp. (Rickettsiales: Ehrlichiaceae). There is strong evidence that these *Wolbachia‐*like bacteria can, although imperfectly (Greiman, Tkach, and Vaughan 2013), spread both horizontally and vertically inside the parasitic worms and successfully infect and divide in the cells of the mammalian hosts (Fischer et al. 2017). As mentioned previously, some species of liver flukes are possible reservoirs of the oncogenic bacteria *H. pylori* , which, with many other factors, are linked to the development of bile duct cancer or cholangiocarcinoma. We argue, however, that the authors miss something very important when they see cholangiocarcinoma as a by‐product of many circumstances. We propose that it is an adaptation of the oncogenic pathogen (Deenonpoe et al. 2017). The fact that the number of bacterial cells and oncogenic virulence cag genes are significantly increased only in host organisms infected with the parasitic worms (Deenonpoe et al. 2017) indicates that cancer induction happens in a functionally adaptive way, not as an unfortunate by‐product. Moreover, fluke infection prevalence was shown to correlate well with the amounts of bacteria and their virulence genes, which further strengthens our adaptive concept of cancer development (Figure 1) (Deenonpoe et al. 2017).

## 4 *Helicobacter pylori* : The Amphibious Pathogen

The widespread notion is that *H. pylori* is a purely extracellular bacterium without an intimate interaction with the host cell; therefore, it needs to inject the oncogenic proteins inside the target cells extracellularly (Hatakeyama 2009). However, new research reveals that these bacteria can reside both extracellularly and intracellularly, which helps them manipulate cell physiology more effectively for their own interests (Huang et al. 2016). To support the adaptive hypothesis of cancer development, we refer to a recent research about the existence of intratumor microbiota. It was a striking scientific result when researchers found a low number of bacterial cells intracellularly inside the tumour which is, according to the study, not entirely sterile and hostile as previously thought (Nejman et al. 2020). Moreover, a Chinese research group found convincing evidence that these microbial residents actively manipulate the behaviour of the affected tumour cells in many different ways (Fu et al. 2022).

## 5 *Helicobacter pylori* Induces Different Types of Cancers Depending on Geography

As previously mentioned, the virulence gene cagA inserted in transgenic mice induces only gastric and mucosa‐associated lymphoid tissue lymphoma (MALT) tumours specifically, without any sign of other types of cancers (Ohnishi et al. 2008). In Southeast Asia, however, cholangiocarcinoma is the typical cancer associated with *H. pylori* linked to flukes (Deenonpoe et al. 2017). Consequently, there might be different adaptive circumstances in Southeast Asia and China, linked to the bacterial pathogens, compared to the temperate regions where the fluke vector is absent.

## 6 How Can We Test These Hypotheses?

We would like to propose some potentially credible tests of our hypothesis.

1. Although we have circumstantial evidence for the tumorigenesis of *H. pylori* , further laboratory experiments could clarify the causative relationship. Within such an experimental setup, cercariae should be treated with antibiotics to eliminate *Helicobacter* infection. Later, these metacercariae should be used to infect a rat/mouse model to test whether the incidence of cancer would be lower in this group than in a control group.
2. It could also be experimentally proven that cholangiocarcinoma has adaptively evolved in endemic tropical regions as a consequence of the mutualistic relationship between flukes and bacteria. In order to provide evidence, the experimental setup of Ohnishi et al. (2008) could be adapted by using cagA oncogenes from *H. pylori* ‐infected *O. viverrini*. By inserting these genes into laboratory mice, we expect to induce only cholangiocarcinoma and not MALT and gastric cancer (Ohnishi et al. 2008).
3. Site‐specific observation could prove that cancer‐inducing flukes in fact feed on the tumour. The exact site of feeding, both in experimental animals and in clinical patients, could be determined by using an endoscope camera.
4. There is evidence that hepatitis B and hepatitis C viruses can also be transmitted by opisthorcids and contribute to cholangiocarcinoma (Ewald and Swain Ewald 2014). Experiments using these viruses in laboratory animals could extend and generalize our hypothesis that trematodes may be vectors for oncogenic pathogens.

## 7 Conclusion

Our hypothesis gives us new perspectives to understand the intriguing medical phenomenon of fluke‐linked cancers. The suggested mechanism for cancer induction provided here may also help us gain more understanding about cancer in general and its relationship with microbes and parasites. Elaborating the unique nexus between flukes, carcinogenic microbes and cancer in the future will help us broaden our oncological perspective to reduce human death and suffering from this serious disease group. This kind of knowledge might be crucial for understanding the pathogenesis of many other cancer types in the future, thereby facilitating the development of novel therapeutics and the identification of patients at increased cancer risk for more focused and timely treatment.

## Conflicts of Interest

The authors declare no conflicts of interest.

## Data Availability

The authors have nothing to report.
